# Intrapulmonary shunting is a key contributor to hypoxia in COVID-19: An update on the pathophysiology

**DOI:** 10.1371/journal.pone.0273402

**Published:** 2022-10-20

**Authors:** Nikhil Mayor, Harry Knights, Aleksandra Kotwica, Andrew Solomon Joseph Coppola, Harriet Hunter, Nathan Jeffreys, Alexander Morgan, Shivani Gupta, James Prentice, Rebecca Macfarlane, Emma Russell-Jones, Theodore Dassios, David Russell-Jones

**Affiliations:** 1 Royal Surrey NHS Foundation Trust, Guildford, Surrey, United Kingdom; 2 Epsom & St Helier NHS Foundation Trust, Epsom, United Kingdom; 3 University College London, London, United Kingdom; 4 Lister Hospital, East and North Hertfordshire NHS Trust, Stevenage, United Kingdom; 5 Guy’s and St Thomas’ NHS Foundation Trust, London, United Kingdom; 6 King’s College London, London, United Kingdom; 7 University of Surrey, Guildford, United Kingdom; Royal College of Surgeons in Ireland, IRELAND

## Abstract

**Background:**

The pathophysiology of COVID-19 remains poorly understood. We aimed to estimate the contribution of intrapulmonary shunting and ventilation-to-perfusion (V_A_/Q) mismatch using a mathematical model to construct oxygen-haemoglobin dissociation curves (ODCs).

**Methods:**

ODCs were constructed using transcutaneous pulse oximetry at two different fractions of inspired oxygen (FiO_2_). 199 patients were included from two large district general hospitals in the South East of England from 1^st^ to 14^th^ January 2021. The study was supported by the National Institute of Health Research (NIHR) Clinical Research Network.

**Results:**

Overall mortality was 29%. Mean age was 68.2 years (SEM 1·2) with 46% female. Median shunt on admission was 17% (IQR 8–24.5); V_A_/Q was 0.61 (IQR 0.52–0.73). Shunt was 37.5% higher in deaths (median 22%, IQR 9–29) compared to survivors (16%, 8–21; p = 0.0088) and was a predictor of mortality (OR 1.04; 95% CI 1.01–1.07). Admission oxygen saturations were more strongly predictive of mortality (OR 0.91, 95% CI 0.87–0.96). There was no difference in V_A_/Q mismatch between deaths (0.60; IQR 0.50–0.73) and survivors (0.61; IQR 0.52–0.73; p = 0.63) and it was not predictive of mortality (OR 0.68; 95% CI 0.18–2.52; p = 0.55). Shunt negatively correlated with admission oxygen saturation (R -0.533; p<0.0001) whereas V_A_/Q was not (R 0.1137; p = 0.12).

**Interpretation:**

Shunt, not V_A_/Q mismatch, was associated with worsening hypoxia, though calculating shunt was not of prognostic value. This study adds to our understanding of the pathophysiology of hypoxaemia in COVID-19. Our inexpensive and reliable technique may provide further insights into the pathophysiology of hypoxia in other respiratory diseases.

## Introduction

Despite the successful deployment of COVID-19 vaccines in the United Kingdom (UK), a proportion of patients continue to present to hospitals with severe hypoxia [1]. A number of treatments including systemic corticosteroids, antivirals, and biological agents are now known to improve outcome [2,3]. A subset of patients, however, still deteriorate and require increasing levels of supplementary oxygen, non-invasive, or mechanical ventilation.

The pathophysiological mechanisms responsible for COVID-19 hypoxaemia remain inconclusive. It is theorised that intrapulmonary shunting (hereafter termed “shunting”) is the primary mechanism by which COVID-19 leads to hypoxia, though development of a shunt is likely multifactorial [4]. The typical physiological response to areas of damaged lung tissue is hypoxic pulmonary vasoconstriction (HPV), reducing perfusion in non-aerated tissue and maintaining the ventilation-perfusion ratio [5]. Evidence from imaging studies suggests that this response may be impaired or even reversed in severe COVID-19 [6]. The shunt may be compounded by microemboli, particularly if these occur in areas of healthy lung, as well as inflammation of the blood-gas barrier leading to diffusion limitation [7]. A greater understanding of the pathophysiology underlying COVID-19 hypoxaemia will guide management approaches and the development of future treatments.

Predicting which patients will require high level care and are at greatest risk of mortality remains challenging. Several studies have identified high-risk demographics such as male sex, age, minority ethnic groups, and low socioeconomic status [8,9]. Cardiovascular and respiratory disease, and immunosuppression, are also predictive of poor outcome [8,10]. Certain laboratory and radiological parameters on admission also have prognostic value: markers of inflammation and hypercoagulability; and the extent of typical “ground-glass” opacification [10]. More specialist parameters such as serum IL-6 and procalcitonin can also be employed with prognostic intent, though at considerable expense [11,12]. Several general and COVID-19 specific composite scoring systems have also been described [13,14]. Nevertheless, identifying which patients are likely to incur a poor prognosis remains challenging, with poor outcomes observed in patients with few or no risk factors. There therefore remains a need for a non-invasive, rapid, and inexpensive triage tool for use in resource-poor settings severely affected by the pandemic, to accurately predict which patients will require higher care.

In a pilot study, we identified shunt as a key contributor to mortality in patients with severe COVID-19, and presented a tool that uses a non-invasive method to estimate shunt [15]. In this study, we aim to validate our findings in a larger, multicentre cohort at the peak of the “second wave” of infections in the UK.

### Aims

1. To identify the contribution of intrapulmonary shunting and V_A_/Q mismatch to hypoxaemia in severe COVID-19
2. To assess whether estimating intrapulmonary shunt by modelling oxygen-haemoglobin dissociation curves from fingertip pulse oximetry in patients with severe COVID-19 is useful in predicting outcome.

## Materials and methods

### Study design and participants

This was a retrospective cohort study performed across two large district general hospitals in the South East of England, with the capacity for level three care, from 1st to 14th January 2021 at the peak of the second wave of COVID-19 in the UK. A power calculation was performed with groups as deaths and survivors and primary outcome as shunt. The anticipated means for groups were 17 (deaths) and 11 (survivors) based on data from our pilot study. This suggested a sample size of 148 was required for an alpha of 0.05 and power of 80%. We considered a 25% drop-out rate due to poor documentation of oxygen saturations on admission. Data from every patient over 18 years old admitted to the two centres for clinically and/or laboratory-confirmed COVID-19 was recorded. Patients were only included if they were hypoxic on admission and required supplemental oxygen. Patients were excluded if they were pregnant, self-discharged against medical advice before treatment completion, or if there was no documentation of oxygen saturations on admission.

### Estimation of intrapulmonary shunt and V_A_/Q mismatch by construction of ODC

The calculation has been previously described in detail. Intrapulmonary shunt and V_A_/Q mismatch were estimated using software based on the algorithm developed by Lockwood et al which produces ODCs using a two-compartment model (S1 Fig) [16]. The method corrects for the concurrent haemoglobin concentration. In principle, intrapulmonary shunting causes a decrease in arterial oxygen saturation due to arteriovenous admixture which cannot be corrected by increasing inspired oxygen. The degree of shunt can therefore be quantified by the degree of depression of the ODC. In contrast, a reduction in V_A_/Q results in reduced oxygen content in post-alveolar blood producing a right shift of the curve which can be overcome by increasing inspired oxygen. These parameters can be quantified by comparing an individual’s ODC to a reference curve which corresponds to a V_A_/Q of 1 (V_A_/Q match) and no right-to-left shunting. Using two values of oxygen saturation measured by pulse oximetry at two different fractions of inspired oxygen (FiO_2_), an ODC was constructed to determine the V_A_/Q ratio and percentage of right-to-left shunt for all patients. In a minority of cases the model produces artificially skewed ODCs giving non-physiological V_A_/Q ratios. This occurs when the two data points are close together on the ODC. V_A_/Q values of up to 2.5 were included for analysis.

### Saturation and FiO_2_ value calculation

Oxygen saturations measured using fingertip pulse oximetry by the ambulance crew and emergency departments were collected. Oxygen flow (litres/min) was converted to FiO_2_ according to predetermined conversion charts [17]. Baseline saturations on air followed by saturations after administration of oxygen were collected and used to construct the ODC.

### Clinical parameters

Epidemiological, clinical, laboratory, and radiological characteristics were collected in addition to treatments and outcome (discharge or death). Smoking was defined as never, current, or ex–cigarette use. All patients underwent a plain chest radiograph within 24 hours of admission reported using the British Institute of Radiology COVID–19 Guidelines and Reporting Templates [18]. The in–hospital deaths included patients placed under palliative care.

### Laboratory measurements

Nasopharyngeal, oropharyngeal, or bronchoalveolar lavage samples were collected from patients for the extraction of SARS-CoV-2 RNA. Commercial isolation kits were used to extract total RNA and real-time PCR assays were performed to achieve qualitative detection of COVID-19 viral RNA. Laboratory investigations including full blood count, renal biochemistry, lactate dehydrogenase (LDH), creatine kinase (CK), ferritin, D-dimer, high sensitivity troponin I, and C–reactive protein (CRP) were measured using routine validated automated clinical assays. Only blood tests performed within 24 hours of admission were included.

### Statistical analysis

Categorical variables are presented as numbers (percentages). Continuous variables are presented as means (± standard error of the mean, SEM) if normally distributed or median (interquartile range, IQR) if not. Continuous data was compared using appropriate statistics for parametric and non-parametric data. Categorical data was compared using Fisher’s exact and Chi squared tests. Spearman and Pearson correlation co-efficients were used to examine the relationship between shunt and V_A_/Q, and clinical and biochemical parameters. P-values below 0.05 were considered to be statistically significant. Univariate logistic regression was performed to explore the association between V_A_/Q ratio, shunt, and arterial oxygen saturations on admission, with the risk of death. Statistical analysis was performed using GraphPad Prism v.8.4.2.

## Results

A total of 211 patients met the inclusion criteria. One patient self-discharged; one was pregnant; and ten did not have oxygen saturations documented. The remaining 199 patients were included in the study. Epidemiological and clinical characteristics are presented in Table 1. Mean age was 68.2 years (SEM 1.2), mean BMI was 29.0 (SEM 0.5), and 46% were female. The median number of days from symptom onset to admission was 7 (IQR 5–10). Common comorbidities were hypertension (47%) and diabetes (21%). COPD (17%) and asthma (17%) were also prevalent. The prevalence of atrial fibrillation (p = 0.0029), ischemic heart disease (p = 0.0035), previous stroke (p = 0.0002), heart failure (p = 0.0004), and chronic kidney disease (p = 0.0023) were higher in the group of patients who died.

**Table 1:** Baseline demographic and clinical parameters.

	Total (n = 199)	Survivors (n = 142)	Deaths (n = 57)	p-value
Mean age, years (±SEM)	68.2 ± 1.2	63.3 ± 1.4	80.2 ± 1.6	**<0.001**
*Sex*				
Male	108	85	23	**0.013**
Female	91	57	34	
*Location*				
Royal Surrey	65	41	24	0.072
St Helier	134	101	33	
*Ethnicity*				
White British	152	104	48	0.099
White Other	11	9	2	0.43
BAME	24	18	6	0.67
Not specified	12	11	1	0.11
*Co-morbidities*				
Hypertension	93	62	31	0.17
Diabetes	42	29	13	0.71
Atrial fibrillation	29	14	15	**0.0029**
Ischaemic heart disease	32	16	16	**0.0035**
Stroke	25	10	15	**0.0002**
Heart failure	21	8	13	**0.0004**
Chronic kidney disease	21	9	12	**0.0023**
Asthma	33	26	7	0.30
COPD	33	22	11	0.51
Current cancer	13	8	5	0.42
Previous cancer	11	9	2	0.43
Smoking	82 (total 188)	57 (total 138)	25 (total 50)	0.29
*Mean Body Mass Index* (±SEM)	29.0 ± 0.5	28.9 ± 0.6	29.4 ± 1.2	0.68
*Vaccination Status*				
Vaccinated	2	1	1	…
Previous COVID-19	4	1	3	…

56% of patients were for full treatment escalation, 19% had a treatment ceiling of non-invasive ventilation (NIV), 25% for ward-based care (i.e. not for admission to intensive care or for treatment with NIV), and 39% had a ‘do not attempt resuscitation’ decision in place. Median length of hospital stay was 6 days (IQR 3–11) (Table 2). There were less deaths in patients who received remedesivir (p = 0.029). There was no difference in survival in patients receiving antibiotics and/or corticosteroids (S2 Table).

**Table 2:** Maximum level of oxygen therapy and outcomes.

Maximum level of therapy	Total (n = 199)	Survivors (n = 142)	Deaths (n = 57)
*Ward-based care*	146	111	35
Oxygen ≤4 L/min	65	61	4
Oxygen >4 L/ min	80	49	31
*NIV*	53	31	22
Median duration of NIV, days (IQR)	5.0 (3.0–6.0)	5.0 (4.0–6.0)	3.5 (2.0–7.5)
*Critical care admission*	35	22	13
Invasive ventilation	10	5	5
Median duration of invasive ventilation, days (IQR)	14.0 (8.2–21.5)	4.5 (3.0–5.7)	10.0 (3.0–17.0)
*Other*			
Median length of stay, days (IQR)	6.0 (3.0–11.0)	6.0 (3.0–11.0)	6.0 (4.0–12.0)

Values are numbers (percentages) unless stated otherwise.

Baseline laboratory tests showed lymphopaenia (median lymphocyte count 0.8 x 10^9^/L, IQR 0.6–1.1) as well as an activated inflammatory response and coagulation cascade (S1 Table). 177 (89%) patients had radiological findings suggestive of COVID-19 pneumonia on plain chest radiographs: 73 (37%) mild; 80 (40%) moderate; and 24 (12%) severe. Severe COVID-19 radiological findings were associated with death (p = 0.014). 46 (23%) underwent CT imaging of the chest. 53 (27%) required NIV and 10 (5%) were intubated. 57 (29%) patients in the overall cohort died. There was no difference in survival between the Black, Asian, and Minority Ethnic (BAME) and non-BAME groups (p = 0.67).

The median oxygen saturation on air on admission was 89% (IQR 86–91), shunt was 17% (IQR 8–24.5), and V_A_/Q was 0.61 (IQR 0.52–0.73) which was lower than the accepted lower bound of 0.8 in the general population [19].

**Fig 1:**
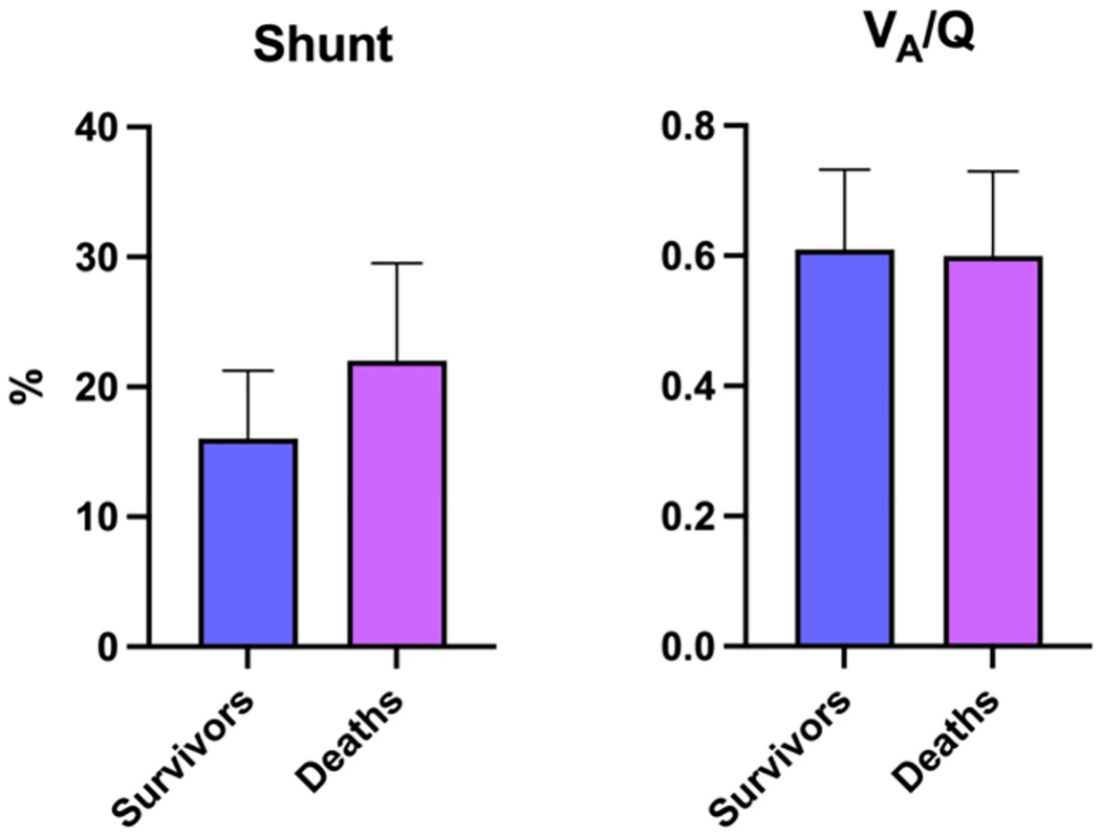
Comparison of admission shunt and V_A_/Q mismatch (median and interquartile range) between deaths and survivors.

Shunt was 37.5% higher in patients who died (22%, IQR 9–29) compared to those who survived (16% IQR 8–21; p = 0.0088) (Fig 1). Shunt was a predictor of mortality upon univariate logistic regression (OR 1.04; 95% CI 1.01–1.07; p = 0.010). There was no difference in shunt (p = 0.22) or V_A_/Q (p = 0.79) in patients with known underlying respiratory chronic pathology such as COPD, asthma, or acutely diagnosed pulmonary embolism during admission, compared to those without underlying respiratory disease. There was no difference in V_A_/Q mismatch between deaths (0.60; IQR 0.50–0.73) and survivors (0.61; IQR 0.52–0.73; p = 0.63) and it was not predictive of mortality upon logistic regression (OR 0.68; 95% CI 0.18–2.52; p = 0.55). Admission oxygen saturations on air were 3.4% lower in patients who died (86%, IQR 80–89) compared to those who survived (89%, IQR 88–91; p<0.00001) and were also predictive of mortality upon logistic regression (OR 0.91, 95% CI 0.87–0.96; p = 0.0003).

Shunt was negatively correlated with admission oxygen saturation (R value -0.533; p<0.0001). V_A_/Q did not correlate with oxygen saturations (R value 0.1137; p = 0.12). Shunt correlated with NEWS2 score on admission (p<0.0001), white cell count (p = 0.0085), neutrophil count (p = 0.016), CRP (p = 0.0003), D-dimer (p = 0.002), LDH (p<0.001) and urea (p = 0.025) (Table 3). It also positively correlated with duration of non-invasive ventilation (p<0.0001), length of hospital stay (p = 0.0075) and number of days on oxygen (p = 0.0025). V_A_/Q mismatch was not related to any of these parameters.

**Table 3:** Correlation of % shunt with clinical parameters.

	Correlation coefficient	*p* value
*Admission characteristics*		
Age	-0.0145	0.84
NEWS2 score	0.315	**<0.0001**
Body Mass Index	0.0213	0.79
*Laboratory findings*		
WCC	0.186	**0.0085**
Neutrophil	0.180	**0.016**
CRP	0.254	**0.0003**
D-dimer	0.3489	**0.002**
LDH	0.506	**<0.001**
Creatine kinase	0.121	0.33
Ferritin	0.0779	0.53
Troponin	0.1800	0.099
Urea	0.16	**0.025**
eGFR	0.015	0.84
*Outcome measures*		
Duration of NIV	0.429	**<0.0001**
Length of hospital stay	0.189	**0.0075**
Oxygen number of days	0.213	**0.0025**
Critical care number of days	-0.220	0.20
Intubation number of days	-0.536	0.24

NEWS2 = National Early Warning Score 2; eGFR = glomerular filtration rate; NIV = non-invasive ventilation.

## Discussion

The pathophysiological mechanisms responsible for hypoxaemia in COVID-19 remain uncertain. However, it is theorised that intrapulmonary shunting and ventilation-perfusion mismatching play important roles. Physiologically, there should be no, or minimal, shunt and the accepted normal value for V_A_/Q is 0.8 and above [19]. In this two-centre study on patients admitted with severe COVID-19, estimated median shunt was 17%, and V_A_/Q ratio was 0.61, and increasing shunt was associated with worsening hypoxia. These findings help to define the pathophysiological mechanisms underlying COVID-19 hypoxaemia. It remains to be determined whether targeting shunt will improve outcome, with data only currently available from a small case series describing the possible beneficial effects of pulmonary vasoconstrictors [20].

Shunt is proposed to occur via impaired HPV secondary to the endogenous release of prostaglandins, bradykinin, and inflammatory cytokines alongside dysregulation of the renin-angiotensin-aldosterone system (RAAS) [21]. Angiotensin-converting enzyme (ACE) receptor activity is reduced after internalisation following viral entry which prevents angiotensin-2 production and vasoconstriction [7]. ACE has also been implicated in an imbalance between procoagulant and fibrinolytic activity responsible for the development of microthrombi [7]. Our findings are consistent with this notion since various inflammatory parameters positively correlated with shunt severity and may therefore have a direct action on pulmonary vasculature.

‘Happy’ or silent hypoxia is a phenomenon commonly described in COVID-19 [22]. It has previously been associated with atelectasis, right-to-left cardiac shunting, and intrapulmonary shunting [7]. It is feasible, therefore, that shunting is involved with the development of silent hypoxia in COVID-19.

Predicting which patients will require high level care and are at greatest risk of mortality remains challenging. We estimated shunt on admission to hospital in a cohort of patients who were relatively early in their disease course. Shunt was 38% higher in patients who died than survived. Furthermore, increased shunt was predictive of mortality and correlated with duration of hospital stay, oxygen therapy, and CPAP. Our findings suggest that estimating shunt on admission for the group of patients with hypoxia secondary to COVID-19 can be used to predict outcome. However, admission oxygen saturations were more strongly predictive of outcome suggesting that the estimation of shunt in patients admitted to hospital with severe COVID-19 provides no additional prognostic value.

Our model is based upon the predictable physiological properties of haemoglobin and therefore provides reliable information in patients in variable clinical situations. This technique makes use of pulse oximetry, a widely available and inexpensive technology, which is able to rapidly and non-invasively derive oxygen-haemoglobin dissociation curves (ODCs) to estimate shunt and V_A_/Q mismatch. Furthermore, in a sensitivity analysis performed comparing those with pre-existing respiratory disease to those without, there was no difference in either shunt or V_A_/Q mismatch. These findings suggest that our model is suitable for detecting shunt in patients with pre-existing respiratory disease and limits any potential confounding effect on shunt in our overall analysis.

This study is limited by its retrospective design with reliance upon clinical judgement for the accurate recording of oxygen saturations after the initiation of oxygen therapy. Furthermore, our technique only provides an estimate of shunt and V_A_/Q mismatching. The direct measurement of intrapulmonary shunting requires invasive monitoring. Therefore, our method provides vital information in patients not requiring critical care, and is translatable to a much larger population than existing data derived from invasive monitoring. Our technique also does not distinguish an intrapulmonary from a right-to-left intracardiac shunt, although this has been described elsewhere [23]. However, controlling for intracardiac shunting would also require invasive imaging. Furthermore, this study took place prior to the widespread deployment of COVID-19 vaccination, as well as the identification of novel variants. Future prospective studies are required to determine whether the relative contributions of shunt and VA/Q mismatch are impacted by vaccination and/or previous infection.

## Conclusion

This study estimates the relative proportions of shunt and V_A_/Q mismatch in people presenting with severe COVID-19 infection. We have shown that intrapulmonary shunt, not V_A_/Q mismatching, is associated with worsening hypoxia. These findings contribute to our understanding of the pathophysiological mechanisms responsible for hypoxia in severe COVID-19. Whilst shunt was predictive of mortality, it did not provide any additional prognostic value beyond simple oxygen saturations on admission.

## Supporting information

S1 Fig Gas exchange model used to construct oxygen-haemoglobin dissociation curves (ODC) for the cohort. Right shift of curve indicates decreasing V/Q ratio, downwards shift indicates increasing shunt.
(TIF)

S1 Table Laboratory findings. (DOCX)

S2 Table Medical treatment of deceased and recovered patients. (DOCX)
