# Supplementary figures and images for: Intrapulmonary shunting is a key contributor to hypoxia in COVID-19: An update on the pathophysiology
Source: PLoS One. 2022 Oct 20;17(10):e0273402. doi: 10.1371/journal.pone.0273402 (PMC9584408; doi:10.1371/journal.pone.0273402)

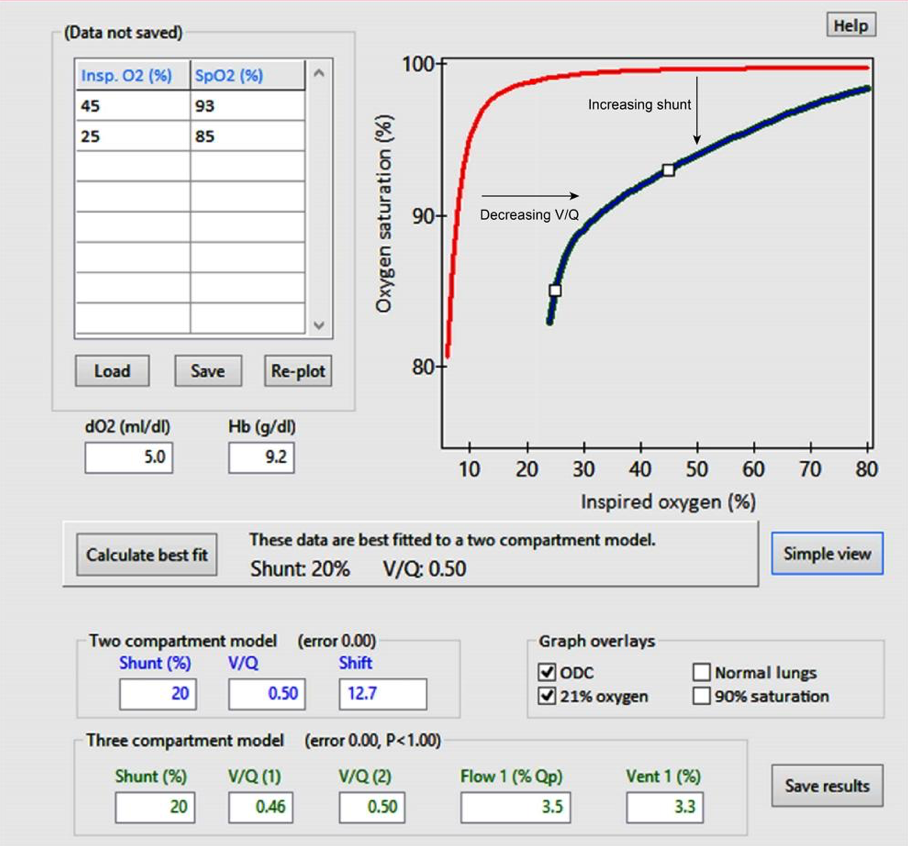

Supplement: S1 Fig — Right shift of curve indicates decreasing V/Q ratio, downwards shift indicates increasing shunt. (TIF) [file pone.0273402.s001.tif]
