# Supplementary material for: Intrapulmonary shunting is a key contributor to hypoxia in COVID-19: An update on the pathophysiology
Source: PLoS One. 2022 Oct 20;17(10):e0273402. doi: 10.1371/journal.pone.0273402 (PMC9584408; doi:10.1371/journal.pone.0273402)
Supplement: S1 Table — (DOCX) [file pone.0273402.s002.docx]

|  | Total  (n=199) | Survivors   (n=142) | Deaths   (n=57) | p-value |
| --- | --- | --- | --- | --- |
| *Laboratory findings (normal range)* | | | | |
| Haemoglobin, g/L (130-180) | 135 (123 – 148) | 137 (125 – 149) | 128 (120 – 144) | **0.033** |
| White blood cell count, 10^9^/L (4-11) | 6.6 (5.1 – 8.8) | 6.6 (5.1 – 8.4) | 6.7 (5.0 – 10.2) | 0.36 |
| Neutrophil count, 10^9^/L (2-7.5) | 5.2 (3.7 – 7.0) | 5.2 (3.7 – 6.5) | 5.2 (3.7 – 9.1) | 0.16 |
| Lymphocyte count, 10^9^/L (1-4) | 0.8 (0.6 – 1.1) | 0.8 (0.6 – 1.1) | 0.7 (0.4 – 0.9) | **0.0016** |
| Platelet count, 10^9^/L (150-450) | 211 (171 – 277) | 221 (176 – 281) | 192 (151 – 264) | **0.028** |
| Sodium, mmol/L (133-146) | 137 (134 – 140) | 137 (134 – 139) | 139 (136 – 141) | **0.0028** |
| Potassium, mmol/L (3.5-5.3) | 4.1 (3.8 – 4.4) | 4.1 (3.8 – 4.4) | 4.1 (3.8 – 4.5) | 0.78 |
| Urea, mmol/L (2.5-7.8) | 6.0 (4.8 – 9.6) | 5.6 (4.5 – 7.4) | 10.5 (6.1 – 17) | **<0.00001** |
| eGFR, mL/min (90-120) | 82 (64 – 90) | 87 (72 – 90) | 54 (38 – 78) | **<0.00001** |
| Creatinine, µmol/L (64-104) | 76 (63 – 98) | 72 (61 – 88) | 100 (68 – 124) | **<0.00001** |
| C-reactive protein, mg/L (0-10) | 100 (59 – 160) | 100 (57 – 156) | 101 (65 – 185) | 0.58 |
| Lactate dehydrogenase, U/L (0-479) | 691 (581 – 889) | 668 (574 – 787) | 860 (693 – 924) | 0.11 |
| Creatine kinase, U/L (40-320) | 194 (90 – 396) | 171 (91 – 387) | 246 (86 – 627) | 0.93 |
| Ferritin, µg/L (15-300) | 916 (459 – 1369) | 1207 (619 – 1517) | 479 (333 – 1236) | **0.011** |
| High-sensitivity troponin I, ng/L (0-46) | 17 (7.0 – 47) | 10 (6 – 28) | 48 (17 – 125) | **<0.00001** |
| D-dimer, ng/mL (0-500) | 1080 (681 – 1818) | 1013 (617 – 1691) | 1368 (845 – 2160) | 0.080 |
