# Supplementary material for: Intrapulmonary shunting is a key contributor to hypoxia in COVID-19: An update on the pathophysiology
Source: PLoS One. 2022 Oct 20;17(10):e0273402. doi: 10.1371/journal.pone.0273402 (PMC9584408; doi:10.1371/journal.pone.0273402)
Supplement: S2 Table — (DOCX) [file pone.0273402.s003.docx]

| Medical treatment | Total  (n=199) | Survivors  (n=142) | Deaths  (n=57) |
| --- | --- | --- | --- |
| Antibiotics | 187 | 134 | 53 |
| Dexamethasone | 194 | 138 | 56 |
| Remdesivir | 76 | 61 | 15 |
| Monoclonal antibodies | 6 | 6 | 0 |
| Colchicine | 3 | 3 | 0 |
| Aspirin | 3 | 3 | 0 |
| Convalescent plasma | 3 | 3 | 0 |
